# Difference in blood pressure response to ACE-Inhibitor monotherapy between black and white adults with arterial hypertension: a meta-analysis of 13 clinical trials

**DOI:** 10.1186/1471-2369-14-201

**Published:** 2013-09-26

**Authors:** Robert N Peck, Luke R Smart, Rita Beier, Anthony C Liwa, Heiner Grosskurth, Daniel W Fitzgerald, Bernhard MW Schmidt

**Affiliations:** 1 Department of Medicine, Bugando Medical Centre, Mwanza, Tanzania; 2 Department of Medicine, Weill Bugando School of Medicine, Mwanza, Tanzania; 3 Center for Global Health, Weill Cornell Medical College, New York, USA; 4 Department of Pediatrics, University Hospital Schleswig-Holstein, Lübeck Campus, Germany; 5 Department of Clinical Pharmacology, Weill Bugando School of Medicine, Mwanza, Tanzania; 6 Department of Epidemiology, London School of Hygiene and Tropical Medicine, London, UK; 7 Department of Nephrology and Hypertension, Hannover Medical School, Hannover, Germany

**Keywords:** Blood pressure, Hypertension, Treatment, ACE-inhibitors, Race, Black, White, Meta-analysis

## Abstract

**Background:**

Among African-Americans adults, arterial hypertension is both more prevalent and associated with more complications than among white adults. Hypertension is also epidemic among black adults in sub-Saharan Africa. The treatment of hypertension among black adults may be complicated by lesser response to certain classes of anti-hypertensive agents.

**Methods:**

We systematically searched literature for clinical trials of ACE-inhibitors among hypertensive adults comparing blood pressure response between whites and blacks. Meta-analysis was performed to determine the difference in systolic and diastolic blood pressure response. Further analysis including meta-regressions, funnel plots, and one-study-removed analyses were performed to investigate possible sources of heterogeneity or bias.

**Results:**

In a meta-analysis of 13 trials providing 17 different patient groups for evaluation, black race was associated with a lesser reduction in systolic (mean difference: 4.6 mmHg (95% CI 3.5-5.7)) and diastolic (mean difference: 2.8 mmHg (95% CI 2.2-3.5)) blood pressure response to ACE-inhibitors, with little heterogeneity. Meta-regression revealed only ACE-inhibitor dosage as a significant source of heterogeneity. There was little evidence of publication bias.

**Conclusions:**

Black race is consistently associated with a clinically significant lesser reduction in both systolic and diastolic blood pressure to ACE-inhibitor therapy in clinical trials in the USA and Europe. In black adults requiring monotherapy for uncomplicated hypertension, drugs other than ACE-inhibitors may be preferred, though the proven benefits of ACE-inhibitors in some sub-groups and the large overlap of response between blacks and whites must be remembered. These data are particularly important for interpretation of clinical drug trials for hypertensive black adults in sub-Saharan Africa and for the development of treatment recommendations in this population.

## Background

Hypertension is a growing problem among black adults worldwide. Black adults have the highest age-adjusted rates of hypertension among all racial groups in the United States of America (USA) and Europe. Among black adults in the USA, for example, 44.4% of men and 43.9% of women have hypertension compared to 33.3% in the general population [1]. Black adults also suffer from increased adverse consequences of hypertension due to: 1) more severe hypertension (>180/110 mmHg), 2) less adequate blood pressure control over the long term, and 3) more comorbid conditions such as diabetes mellitus and chronic kidney disease [2].

In sub-Saharan Africa, hypertension is epidemic among black adults. Although previously considered rare [3-6], the prevalence of hypertension in sub-Saharan Africa is rising rapidly due to the effects of urbanization and industrialization on diet, exercise and obesity [3,4,7,8]. In a recent large community-based study in Tanzania, for example, 21% of black adults between the ages of 35–44 years had hypertension; of these, only 18% were aware of their diagnosis, only 14% were on treatment, and only 5% were controlled [8]. Several studies among black adults in Nigeria and South Africa have consistently reported that 15-20% of all hospital admissions in Africa are due to hypertension-related diagnoses [9-12]. Autopsy and death certificate studies show that a large proportion of in-hospital deaths are hypertension-related even among younger adults [13-16].

One frequently cited challenge of treating hypertension among black adults is the lesser blood pressure response to certain medications such as ACE-inhibitors (ACEI) [17-20]. The objectives of this meta-analysis were to combine all currently available evidence from clinical trials of ACEIs to identify the following: 1) if a significant difference in blood pressure response to ACEIs exists between black and non-black populations, 2) the magnitude of this potential difference and 3) whether this potential difference may be explainable by factors other than race.

## Methods

We systematically searched PubMed, EMBASE and Web of Science for any prospective clinical trials providing race specific data on blood pressure lowering during treatment with ACEIs for adults with arterial hypertension on 3 August 2012. The search strategies were created by practicing clinicians and a research librarian and are provided in the Additional file 1: Table S1. No publication date or publication status restrictions were applied, and all languages were allowed. In addition, reference lists of review articles and the selected articles were searched for additional sources.

Once the systematic literature search was complete, two independent reviewers appraised all articles according to a standard set of inclusion and exclusion criteria. In cases of differing results, a third reviewer adjudicated. In the first step, articles were excluded or included based on a review of their titles and abstracts. The remaining articles were then evaluated in full text. All articles fulfilling the below-mentioned criteria were selected and data were extracted.

Inclusion/exclusion criteria were as follows. The study must be a prospective clinical trial providing race specific data on blood pressure lowering during treatment with ACEI. Study participants must be aged ≥18 years. Studies were included only if they enrolled patients for treatment of confirmed arterial hypertension. In order to evaluate the pure effect of ACEI therapy itself on blood pressure reduction, studies that used combination therapy were excluded from the analysis. The minimum treatment duration was 4 weeks; the minimum patient number was ten.

Two independent reviewers performed data extraction in duplicate. In the case of disagreement, a 3rd reviewer examined the article in order to correct the difference. Data were all entered into a standardized evidence table (see Additional file 2: Table S2). The dual primary metameters and measures of variance in this meta-analysis were change in systolic and diastolic blood pressure in mmHg and standard deviation from before start of ACEI treatment to the end of the study period.

The following parameters were also extracted and included in the evidence table: number of patients in each race group, Jadad score (for quality), average age, age difference between races, gender distribution, ACE-inhibitor used and average dose, time interval between start of treatment and blood pressure measurement. Of note, the Jadad score is a standard measure of the quality of clinical trials ranging from 0 (low quality) to 5 (high quality) that takes into account the following components: randomization, blinding, and loss to follow-up [21]. ACE-inhibitor doses were standardized as "enalapril adjusted doses". The doses of fosinopril, quinapril, benazepril and lisinopril were identical to those of enalapril; the trandolapril dose was multiplied by 10; the ramipril dose was multiplied by 4; perindopril doses of 2, 4, 6, and 8 mg and captopril daily doses of 18.75, 37.5, 75, and 150 mg were equivalent to enalapril doses of 5, 10, 20, and 40 mg.

For one study, two references were used for data extraction [22,23]. For that study, the authors were contacted in order to get the standard deviations of the mean blood pressures. ImageJ (free share software, NIH, USA) was used in two articles to obtain measures of mean and standard deviation from a graph.

The meta-analysis was performed using random effects model. Mean difference and 95% confidence intervals (95% CI) were calculated. We tested for heterogeneity by Q test and I^2^ statistics. We assessed possibility of publication bias by evaluating a funnel plot for asymmetry and by Egger’s regression intercept. To evaluate the possible sources of heterogeneity we performed metaregression and stratification according to the above-mentioned factors. As two studies in the meta-analysis had a much larger sample sizes than the other studies, we performed a one-study-removed analysis to assess whether the removal of any one study changed the overall results of the meta-analysis.

We hypothesized that black race was associated with a lesser reduction in systolic and diastolic blood pressure in response to ACE-inhibitor therapy. We also hypothesized that possible sources of heterogeneity between studies could include: age, ACE-inhibitor dose, study quality (i.e. Jadad score), treatment duration and/or baseline blood pressure.

## Results

**Figure 1:**
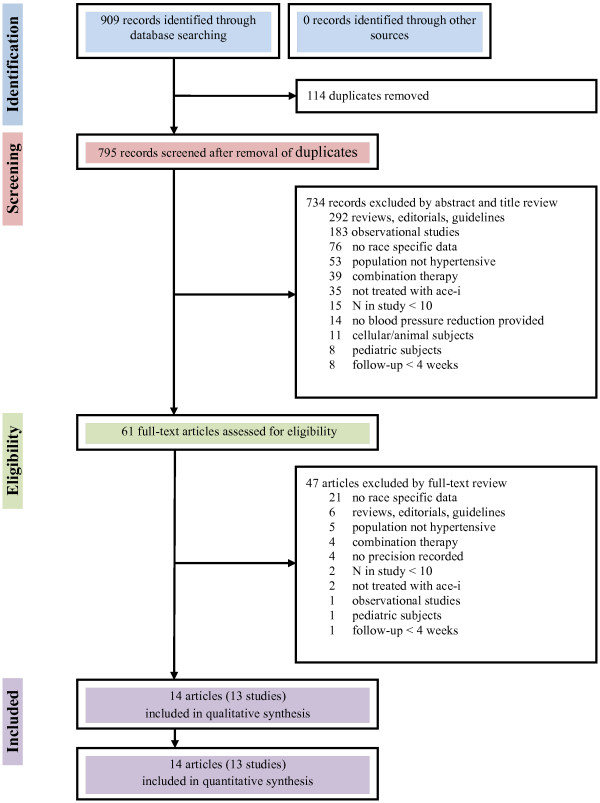
Flow diagram for identification, screening, and inclusion of papers for this meta-analysis.

Our systematic literature search revealed 909 articles: 252 from PubMed, 137 from Embase and 520 from Web of Science. After removal of duplicates, 795 articles were reviewed on the basis of the title and the abstract. This led to the removal of 734 articles. The 61 remaining articles were examined in full text. Of these, 14 fulfilled the inclusion/exclusion criteria. These 14 articles reported 13 different studies and provided 17 different patient groups for evaluation [22-35]. The main reasons for exclusion are described in Figure 1.

Table 1 shows the characteristics of the 13 clinical trials included in our meta-analysis. Studies recruited between 25 and 8887 patients and were conducted between 1982 and 2009. All but one [34], were conducted in the USA. The mean age ranged from 47.5 to 66 years and was typical for hypertensive cohorts. A large variety of ACEIs across the recommended dose range were used. See Additional file 2: Table S2 for details regarding study designs and type of ACEI. Table 2 shows the findings of these 13 clinical trials.

**Table 1:** Characteristics of the 13 clinical trials (17 patient groups) included in this meta-analysis

		**N of subjects**		**Age of subjects (years)**		**Duration (weeks)**	**Jadad score**	**Mean baseline blood pressure systolic/diastolic (mm Hg)**	
		**White**	**Black**	**White**	**Black**			**White**	**Black**
1	Study Group 1982 [26]	170	151	55		7	3	148.2/97.4	147.6/97.8
2	Weinberger 1985 [27]	32	37	*	*	6	0	149.5/99.6	152.7/99.2
3	Thind 1988 [28]	15	10	47.5		4	0	*/97	*/101
4a	Materson 1993, young [29]	39	44	51	49	4	5	149/99	147/100
4b	Materson 1993, old [29]	55	48	66		4	5	154/98	157/100
5a	Weir 1995, 1 mg [24]	51	23	58.3	52.7	6	5	154.8/100.3	149.6/100.2
5b	Weir 1995, 2 mg [24]	53	22	57.3	53.0	6	5	151.9/101.7	144.8/100.2
5c	Weir 1995, 4 mg [24]	53	23	53.0	53.2	6	5	147.6/99.9	155.9/102.9
6	Chrysant 1996 [30]	30	10	53.7		6	4	*	*
7	Weir 1998a [32]	32	13	51		12	5	145.6/89	145.8/91.2
8a	Weir 1998b, 20 mg [31]	48	22	55.5	51.2	4	3	159.9/103	156/105.8
8b	Weir 1998b, 40 mg [31]	43	19	55.6	51.2	4	3	158.4/102.7	157.1/104.8
9	Pahor 2002 [25]	15	18	59.5		4	5	140/86	144/86
10	Cohn 2004 [23], Julius 2004 [22]	7745	1412	56.8	52.3	12	1	157/94.1	156.3/96.5
11	Mokwe 2004 [33]	2046	533	48.9	51.9	18	0	152.2/94.9	150.9/95.6
12	Moran 2007 [35]	89	72	53.8	54.0	8	1	147/87	151/88
13	Van Rijn-Bikker 2009 [34]	33	48	48		6	3	153/95	156/99

*Results not reported.

**Table 2:** Findings from the 13 clinical trials (17 patient groups) included in this meta-analysis

		**Decrement in blood pressure with drug treatment (mm Hg)**									
		**Systolic**					**Diastolic**				
**Trial**	**Standardized mean dose (mg/d)**	**White mean**	**Black mean**	**White-black difference**	**White SD**	**Black SD**	**White mean**	**Black mean**	**White-black difference**	**White SD**	**Black SD**
1	20	14.7	9.1	5.6	15.6	14.7	10.7	7.9	2.8	7.8	8.6
2	20	10.8	0.7	10.1	16.4	3.6	9	4.1	4.9	9.1	11.6
3	5	*	*	*	*	*	8	1.5	6.5	15.1	11.7
4a	16	11	8	3	9	11	10	9	1	7	7
4b	16	11	7	4	9	12	11	7	4	5	7
5a	10	12.8	2.7	10.1	15	13.9	6.1	2	4.1	4.3	4.8
5b	20	9.6	2.5	7.1	15	13.1	8.1	3.8	4.3	5.1	5.2
5c	40	9.1	11.9	-2.8	13.8	13.4	8.9	6.5	2.4	4.4	5.3
6	20	*	*	*	*	*	11.8	6.2	5.6	7.8	10
7	24	14.1	7.8	6.3	10.2	14.4	10.2	6.2	4	6.2	11.5
8a	20	13.4	8.5	4.9	16.3	12.9	9.8	7.3	2.5	8.1	7.5
8b	40	16.8	12.3	4.5	18.5	20	12	10.1	1.9	8.5	11.3
9	30	5.9	9.2	-3.3	13.7	11.9	2.2	3.1	- 0.9	8.1	11.7
10	18	18.2	14.3	3.9	16.3	17.3	10.6	9	1.6	9.6	10.5
11	*	15.3	10.6	4.7	12.2	13.4	9.8	7.4	2.4	7.8	7.8
12	40	11	5	6	14.2	15.3	7	3	4	7.8	8.7
13	15	9.5	0.9	8.6	15.8	19.9	*	*	*	*	*

SD indicates standard deviation. *Results not reported.

**Figure 2:**
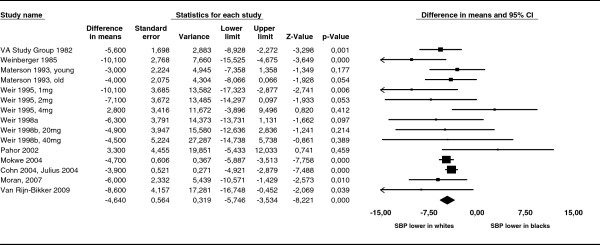
**Forest Plot for difference in mean reduction of systolic blood pressure (SBP).** Forest Plot for difference in mean reduction of systolic blood pressure (SBP) between whites and blacks. White race was associated with a greater reduction in SBP (mean difference: 4.64 (95% CI 3.53-5.75)).

Data on systolic blood pressure changes were available from 15 patient groups out of 11 studies. In the pooled analysis, white race was associated with a greater reduction in systolic blood pressure (mean difference 4.64 mmHg (95% CI 3.53 - 5.75)). We detected little heterogeneity within this comparison. The I^2^ statistic was 24.2 and the Q test was not statistically significant (p = 0.19). The Forest Plot for difference in mean change in systolic blood pressure between whites and blacks is displayed in Figure 2.

**Figure 3:**
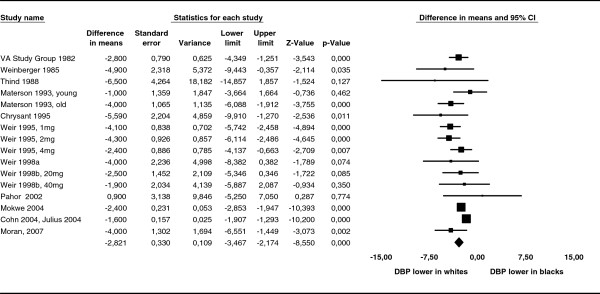
**Forest Plot for difference in mean reduction of systolic blood pressure (SBP).** Forest Plot for difference in mean reduction of diastolic blood pressure (DBP) between whites and blacks. White race was associated with a greater reduction in DBP (mean difference: 2.82 (95%CI 2.17-3.47)).

Data on diastolic blood pressure changes were available from 16 patient groups out of 12 studies. In the pooled analysis, white race was associated with a greater reduction in diastolic blood pressure (mean difference 2.82 mmHg (95% CI 2.17 – 3.47)). We detected some heterogeneity within this comparison. The I^2^ statistic was 59.0 and the Q test was statistically significant (p < 0.0001). The Forest Plot for difference in mean change in diastolic blood pressure between whites and blacks is displayed in Figure 3.

**Figure 4:**
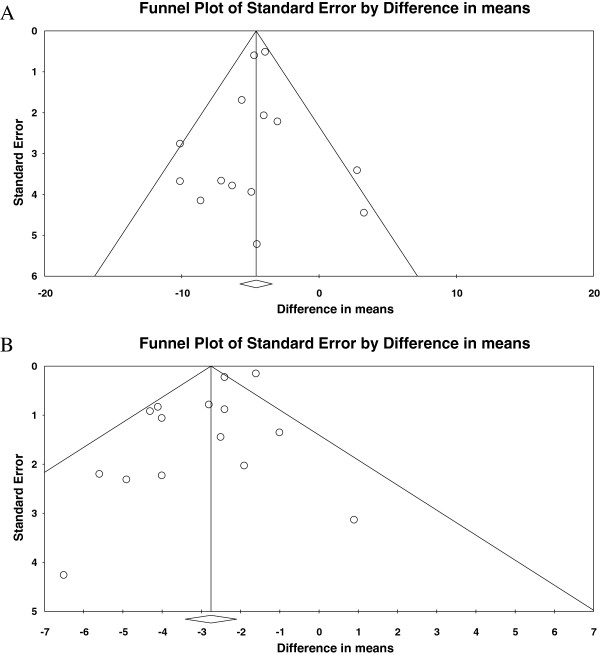
**Funnel Plots for standard error of changes in systolic (A) and diastolic (B) blood pressure.** Funnel Plots for standard error of difference in mean change in systolic **(A)** and diastolic **(B)** blood pressures. These Funnel Plots do not show any evidence of publication bias.

In order to detect potential publication or reporting bias, we performed a separate Funnel Plot analysis for systolic and diastolic blood pressure. Figure 4 shows the Funnel Plots, which did not suggest relevant publication bias. The Eggers Statistics supported this conclusion for the systolic blood pressure (p = .33), whereas it was statistically significant for diastolic blood pressure (p = 0.01).

**Figure 5:**
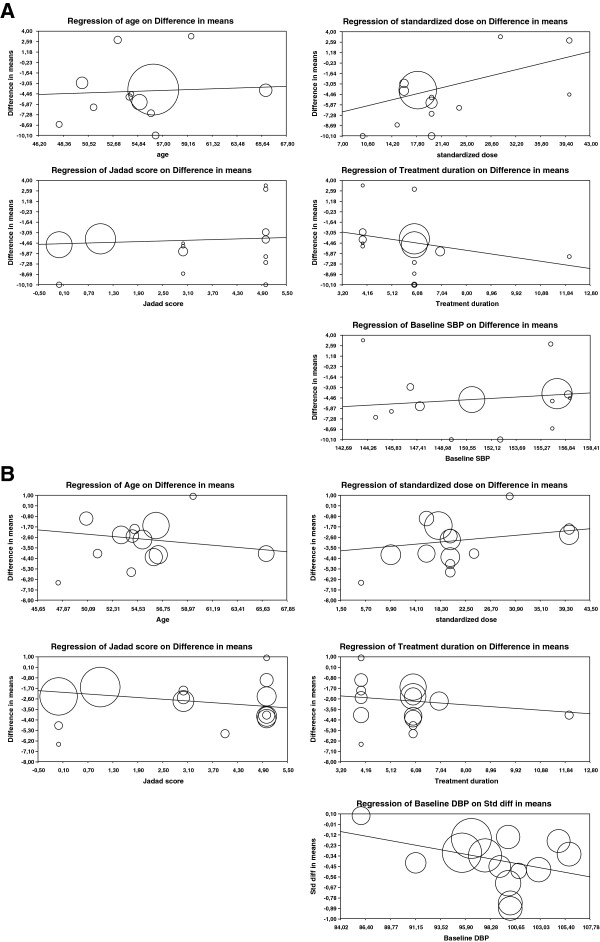
**Meta-regression analyses for systolic (A) and diastolic (B) blood pressure. (A)** Meta-regression analyses for systolic blood pressure (SBP). The difference in SBP reduction between whites and blacks was significantly blunted by increasing doses of ACE inhibitors (p = 0.048). None of the other associations were statistically significant. Meta-regression analyses for diastolic blood pressure (DBP). **(B)** Meta-regression analyses for diastolic blood pressure (DBP). None of the associations were statistically significant but there was a trend toward lesser difference in DBP reduction between whites and blacks when greater doses of ACE-inhibitors were used.

We performed meta-regression analysis of age, ACEI dosage, duration of therapy, Jadad score and baseline blood pressure with regard to systolic (Figure 5A) and diastolic (Figure 5B) blood pressure changes. For systolic blood pressure, use of higher doses of ACEIs was associated with a statistically significant lesser difference in mean blood pressure reduction between whites and blacks (p = 0.048). There was also a trend toward the same finding in diastolic blood pressure but this was not statistically significant. None of the other meta-regressions were statistically significant and most other meta-regression variables trended toward even greater difference in mean reduction in blood pressure with greater age, duration of treatment, study quality, and baseline blood pressure.

**Figure 6:**
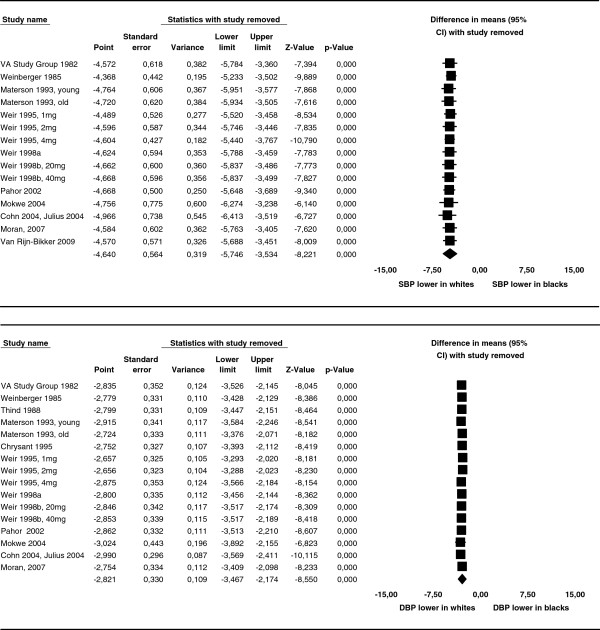
**One-study-removed analysis for difference in mean reduction of systolic and diastolic blood pressures.** One-study-removed analysis for difference in mean reduction of systolic (SBP, above) and diastolic (DBP, below) blood pressure between whites and blacks. Removal of each single study did not change the result of the meta-analysis.

As two studies in the meta-analysis had much larger sample sizes than the other studies, we performed a one-study-removed analysis to assess whether the removal of any one study changed the overall results of the meta-analysis (Figure 6). No change was observed.

## Discussion

In this meta-analysis of 13 unique clinical trials of ACEI therapy for hypertensive adults providing race-stratified systolic and/or diastolic blood pressure results, black race was associated with a consistently lesser response in both systolic and diastolic blood pressure after ACEI therapy (4.6 and 2.8 mmHg respectively). The observed differences in blood pressure response are not only statistically significant but also clinically important since a reduction of 4–5 mmHg in SBP and 2–3 mmHg in DBP has been associated with a 8-20% reduction in risk of cardiovascular morbidity and mortality [36]. This difference in BP response is also similar to the hypotensive effect of some classes of anti-hypertensive agents such as beta-blockers [37]. These results provide evidence for recent recommendations that ACEI should not preferred for monotherapy in black adults with uncomplicated hypertension [2,19], and that calcium channel blockers and thiazide diuretics may be a better choice [38]. These data have particularly important public health implications for black adults with hypertension in sub-Saharan Africa where hypertension is epidemic and guidelines for the treatment of hypertension must consider cost, availability and population-level, average blood pressure responses [3,4,7,8].

The difference in blood pressure response to ACEI monotherapy between blacks and whites observed in this meta-analysis appears to be related to race and is not entirely explainable by other factors. One-study-removed analysis and funnel plots showed no evidence of overinfluence by a single study or of publication bias. We also performed careful metaregression to estimate the impact of possible confounders such as age, baseline blood pressure, duration of treatment, study quality and ACEI dose. Of these factors, only ACEI dose had some mitigating effect on the difference in blood pressure response. Higher doses of ACEI may be beneficial among black adults who require ACEI therapy for hypertension although higher doses may be associated with more adverse effects. One additional, possible confounder in the relationship between race and ACE-inhibitor response is salt intake. Three of the studies included in this meta-analysis investigated the impact of salt intake on the relationship race and ACE-inhibitor with mixed results [31,34,35]. The most rigorous of these studies demonstrated that salt-sensitive black adults had a consistently lesser blood pressure response to ACE-inhibitors whether the subjects were consuming a high or low salt diet. It also showed that a low salt diet was associated with less response to ACEIs among all races [31].

The magnitude of the difference in blood pressure response to ACEI therapy observed in this meta-analysis is quantitatively similar to that observed in other literature. In the largest randomized, controlled trial that compared outcomes to ACEI therapy among black and white adults with hypertension (the ALLHAT study), the differences in systolic and diastolic blood pressure response to lisinopril were 5.6 and 2.5 mmHg respectively [39], almost identical to the differences seen in our meta-analysis. The ALLHAT study was not included in this meta-analysis, though, because many participants received combination therapy and no data for those patients receiving monotherapy were extractable from trial publications. These differences are also similar to the results of 2 prior, smaller meta-analyses of ACEI response in blacks and whites [38,40].

It is important to note that the variability of response to ACEI monotherapy is greater within races than between races. As seen in Table 2, the standard deviation for blood pressure responses among whites and blacks was consistently greater than the difference between these 2 groups. These results affirm the meta-analysis of Sehgal, which showed that the percentage of whites and blacks with similar drug-associated changes in diastolic blood pressure was 90% for diuretics, 90% for beta-blockers, 95% for calcium channel blockers, and 81% for ACEI [40]. Clearly we are, as races, more similar than we are different.

Both the USA JNC-7 guidelines and the UK NICE guidelines have noted the difference in in ACEI response between white and black adults [41,42]. According to the NICE guidelines, calcium channel blockers are preferred over ACE-inhibitors for monotherapy of uncomplicated hypertension in black adults of any age and in white adults over the age of 55 years [41]. The cutoff of 55 years is an interesting one. Of the studies included in this meta-analysis only 1 looked at the difference in blood pressure response to ACE-inhibitors between blacks and white in different age groups. In this study, the systolic and diastolic blood pressure response to ACEI therapy was similar in younger vs. older whites and younger vs. older black adults and was consistently less in younger and older blacks vs. younger and older whites [29]. The recommendation in the NICE guidelines to prefer calcium channel blockers is also notable and further meta-analysis is needed to better define the relationship between race and blood pressure response to calcium channel blockers.

These findings must also be taken in the context of other clinical trials that have demonstrated the benefit of ACEI therapy among blacks with certain complications such as chronic kidney disease and diabetes mellitus despite lesser reduction in blood pressure [43]. Also, use of ACEIs as part of a combination for treatment of hypertension has proven to be effective among black adults [2]. In fact, according to one meta-analysis, combination therapy including ACEIs may actually result in a greater blood pressure reduction in black adults than white adults [38]. Since black adults are disproportionately affected by heart and kidney disease and many will require at least 2 hypertensive agents for blood pressure control, the use of a combination therapy including an ACE-inhibitor or angiotensin receptor blocker may be particularly beneficial in this population.

This meta-analysis has several important limitations. First, all of the studies included in this meta-analysis except one were done in the USA and therefore may not be applicable to black adults living in other regions such as sub-Saharan Africa. Although this is a limitation, we also believe that this meta-analysis is important in providing context for the much-anticipated results of the first large, randomized, controlled trials of antihypertensive drugs among hypertensive black adults in sub-Saharan Africa [44]. Also, this meta-analysis was performed on pooled study results and not individual level data. The consistency of the difference in mean blood pressure reduction, however, does point to a real difference in reduction by race. Finally, as with any meta-analysis, this study is limited by the variables that were studied and reported in the component trials. Since hypertension is multifactorial, some important confounders such as nutrition, salt intake and handling, nephron number or hormone levels may have been missed.

## Conclusion

In conclusion, this study confirms and quantifies the hypothesis that adult black hypertensive patients do generally have a lesser mean reduction in systolic and diastolic blood pressure response to ACEI monotherapy when compared to whites. This meta-analysis provides evidence for current guidelines that recommend that drugs other than ACEI should be preferred for uncomplicated, mild-moderate hypertension in blacks, guidelines that have particular import in sub-Saharan Africa. These results, though, must be taken in the context of other research that provides strong evidence for the benefits of ACEIs in some subgroups of hypertensive black adults, particularly those with certain complications such as chronic kidney disease and diabetes mellitus and those requiring multi-drug therapy for their hypertension. For hypertensive black adults in whom ACEI monotherapy is indicated, higher doses may be necessary to obtain the desired reduction in blood pressure.

## Abbreviations

ACEI: Angiotensin converting enzyme inhibitors; DBP: Diastolic blood pressure; SBP: Systolic blood pressure; UK: United Kingdom; USA: United States of America.

## Competing interests

The authors declare that they have no competing interests.

## Authors’ contributions

RNP conceived of the study, participated in its design and coordination and drafted the manuscript. LRS coordinated data collection and drafted the manuscript. RB participated in the design of the study and assisted in data collection. ACL drafted the manuscript. HG assisted in the analysis and revised the manuscript. DF assisted in the analysis and revised the manuscript. BMWS conceived of the study, participated in its design, and performed the analysis. All authors read and approved the final manuscript.

## Pre-publication history

The pre-publication history for this paper can be accessed here:

http://www.biomedcentral.com/1471-2369/14/201/prepub

## Supplementary Material

Additional file 1: Table S1 Search Strategies. Click here for file

Additional file 2: Table S2 Evidence Table (after removing data already included in Tables 1 &2). Click here for file
