# Supplementary material for: Difference in blood pressure response to ACE-Inhibitor monotherapy between black and white adults with arterial hypertension: a meta-analysis of 13 clinical trials
Source: BMC Nephrol. 2013 Sep 26;14:201. doi: 10.1186/1471-2369-14-201 (PMC3849838; doi:10.1186/1471-2369-14-201)
Supplement: Additional file 1: Table S1 — Search Strategies. [file 1471-2369-14-201-S1.doc]

**Additional file 1: Table S1** Search Strategies

| Database | Search Terms |  |
| --- | --- | --- |
| Pubmed | ("Angiotensin-Converting Enzyme Inhibitors"[Mesh] OR "Angiotensin-Converting Enzyme Inhibitors"[Pharmacological Action]) AND ("Ethnic Groups"[Mesh] OR "Continental Population Groups"[Mesh]) AND ("Hypertension"[Mesh] OR "Blood Pressure"[Mesh]) | 252 articles  3 Aug 2012 |
| EMBASE | 'hypertension'/exp OR 'blood pressure'/exp AND 'ethnic or racial aspects'/exp AND 'dipeptidyl carboxypeptidase inhibitor'/exp AND [randomized controlled trial]/lim | 137 articles  3 Aug 2012 |
| Web of Science | (TS=((ethnic* OR race OR cultur* OR Cauca* OR Africa* OR White OR Black) AND (patient* ORsubject* OR cohort OR participant*)) AND (TS=(Hypertension) OR TS=(Blood NEAR/3 pressure)) AND TS=((ACE NEAR/3 Inhibitors) OR (Angiotensin NEAR Inhibitors)) AND TS=(trial* OR random* OR clinical OR control* OR RCT)) | 520 articles  3 Aug 2012 |
| TOTAL |  | Total 909 articles (WITH 114 duplicates) |
