# Supplementary material for: Difference in blood pressure response to ACE-Inhibitor monotherapy between black and white adults with arterial hypertension: a meta-analysis of 13 clinical trials
Source: BMC Nephrol. 2013 Sep 26;14:201. doi: 10.1186/1471-2369-14-201 (PMC3849838; doi:10.1186/1471-2369-14-201)
Supplement: Additional file 2: Table S2 — Evidence Table (after removing data already included in Tables 1 &2). [file 1471-2369-14-201-S2.doc]

**Additional file 2: Table S2 Evidence Table (after removing data already included in Tables 1 & 2)**

|  |  |  |  |  |  | Sex (% male) | |  |  |
| --- | --- | --- | --- | --- | --- | --- | --- | --- | --- |
|  |  | Drug | Mean Dose (mg/day) | Dose Titration | Mode of Measurement | White | Black | Multicenter | Country |
| 1 | Study Group 1982 [26] | captopril | 97.5 | No | manual | * | | Yes | USA |
| 2 | Weinberger 1985 [27] | captopril | 75 | No | * | * | | No | USA |
| 3 | Thind 1988 [28] | enalapril | 5 | No | manual | * | | No | USA |
| 4a | Materson 1993, young [29] | captopril | 59.3 | Yes | manual | 100 | | Yes | USA |
| 4b | Materson 1993, old [29] | captopril | 59.3 | Yes | manual | 100 | | Yes | USA |
| 5a | Weir 1995, 1mg [24] | trandolapril | 1 | No | manual | 65 | 30 | Yes | USA |
| 5b | Weir 1995, 2mg [24] | trandolapril | 2 | No | manual | 68 | 45 | Yes | USA |
| 5c | Weir 1995, 4mg [24] | trandolapril | 4 | No | manual | 53 | 35 | Yes | USA |
| 6 | Chrysant 1996 [30] | benazepril | 20 | No | manual | 67 | | Yes | USA |
| 7 | Weir 1998a [32] | lisinopril | 24 | Yes | ambulatory | 53 | | Yes | USA |
| 8a | Weir 1998b, 20mg [31] | enalapril | 20 | Yes | manual | 63 | 51 | Yes | USA |
| 8b | Weir 1998b, 40mg [31] | enalapril | 40 | Yes | manual | 62 | 51 | Yes | USA |
| 9 | Pahor 2002 [25] | fosinopril | 30 | No | manual | 75 | | No | USA |
| 10 | Cohn 2004 [23], Julius 2004 [22] | perindopril | 5.6 | Yes | * | 50 | 44 | Yes | USA |
| 11 | Mokwe 2004 [33] | quinapril | * | Yes | manual | 49 | 39 | Yes | USA |
| 12 | Moran 2007 [35] | Ramipril | 10 | No | ambulatory | 63 | 47 | Yes | USA |
| 13 | Van Rijn-Bikker 2009 [34] | lisinopril | 15 | Yes | automated | 59 | | No | Netherlands |
| *Results not reported. | | | | | | | | | |
